# Towards Neuronal Organoids: A Method for Long-Term Culturing of High-Density Hippocampal Neurons

**DOI:** 10.1371/journal.pone.0058996

**Published:** 2013-04-25

**Authors:** George K. Todd, Casey A. Boosalis, Aaron A. Burzycki, Michael Q. Steinman, Lynda D. Hester, Pete W. Shuster, Randen L. Patterson

**Affiliations:** 1 Department of Physiology and Membrane Biology, School of Medicine, University of California Davis, Davis, California, United States of America; 2 Department of Biochemistry, School of Medicine, University of California Davis, Davis, California, United States of America; 3 The Center for Translational Bioscience and Computing, University of California Davis, Davis, California, United States of America; 4 Molecular, Cellular and Integrative Physiology Graduate Group, University of California Davis, Davis, California, United States of America; 5 Department of Psychology, University of California Davis, Davis, California, United States of America; 6 The Solomon H. Snyder Department of Neuroscience, Johns Hopkins University, Baltimore, Maryland, United States of America; 7 Neuromics, Edina, Minnesota, United States of America; Federal University of Rio de Janeiro, Brazil

## Abstract

One of the goals in neuroscience is to obtain tractable laboratory cultures that closely recapitulate *in vivo* systems while still providing ease of use in the lab. Because neurons can exist in the body over a lifetime, long-term culture systems are necessary so as to closely mimic the physiological conditions under laboratory culture conditions. Ideally, such a neuronal organoid culture would contain multiple cell types, be highly differentiated, and have a high density of interconnected cells. However, before these types of cultures can be created, certain problems associated with long-term neuronal culturing must be addressed. We sought to develop a new protocol which may further prolong the duration and integrity of E18 rat hippocampal cultures. We have developed a protocol that allows for culturing of E18 hippocampal neurons at high densities for more than 120 days. These cultured hippocampal neurons are (i) well differentiated with high numbers of synapses, (ii) anchored securely to their substrate, (iii) have high levels of functional connectivity, and (iv) form dense multi-layered cellular networks. We propose that our culture methodology is likely to be effective for multiple neuronal subtypes–particularly those that can be grown in Neurobasal/B27 media. This methodology presents new avenues for long-term functional studies in neurons.

## Introduction

One goal that cell-biologists seek to achieve is cellular cultures that closely recapitulate physiologically relevant organ systems, commonly termed organoids. These types of cultures have advantages due to their mixed cellular populations which can form differentiated structures *in vitro* [1]–[3]. One of the challenges in creating these organoids is culturing cells for long periods of time (weeks to months). As a prerequisite to creating neuronal organoids, long-term culturing conditions for dense neuronal populations must be optimized. This is particularly challenging, as dense long-term cultures are often not possible, due to glial proliferation, contamination, and/or improper nutrient supplementation. Therefore, developing better culturing techniques that can maintain viable neuronal cultures for longer periods of time is necessary to explore more developed neural systems.

A series of benchmarks are used by experimentalists to ensure viable cultures including: (i) dense synapses, (ii) activity, (iii) connectivity, and in some cases (iv) protein expression (e.g. MAP2, Synapsin, Tau, etc.). The hippocampus is particularly relevant to long-term culturing experiments when considering the enhanced neurogenerative capacity associated with this area of the brain and memory formation [4], [5]. However, previous studies that focused on creating long-term hippocampal cultures met with limited success [6], [7]. In past years, most neuronal cultures have been prepared in the presence of Cytosine β-D-arabinofuranoside (Ara-C) that contained small numbers of glia, so as not to contaminate the neuronal population. Recent studies have demonstrated that neurons require glia for proper formation, connectivity, and activity. In particular, the long-term culture studies performed by Bertrand et al. (2011) and Majd et al. (2008) demonstrated that E18 rat hippocampal cultures could be maintained up to 45 days; however, after this span the neurons experienced significant degradation [6], [7]. Considering the available data, it becomes clear that a more successful method would not only maintain the neurons for longer periods of time, but would also contain a sufficiently large, but controlled glial population, so that proper neuronal morphology and physiology may develop *in vitro*.

Neuronal harvesting has been reduced to common practice such that industrial resources now exist for obtaining neurons ready for use. However, plating and maintaining these cultures still proves to be problematic. Furthermore, while the Banker method [8], and other methodologies that closely resemble it, are excellent for producing low-density pure neuronal cultures, methods for producing long-term co-cultures (for months at a time) are lacking. Co-cultures have the advantage of producing tripartite synapses with glia [9]. Indeed, 50% of synapses in the mature hippocampus are in direct contact with astrocytes, the predominating cell type in glial populations of this tissue [10].

Considering the limitations mentioned above, we here present an optimized, hybrid method for maintaining long-term hippocampal cultures–one that incorporates key elements of the Banker method along with the addition of other improved methodologies which were determined empirically. This newly proposed method can produce tens of cultures from a single dissection and provides alternative solutions to the problems associated with traditional neuronal cultures. Moreover, we determined that this method allows viable neurons in high densities to be grown for at least 120 days, forming large-scale neuronal circuits. We observe that cultures mature over time, and in some cases mirror functional results obtained from *in vivo* studies such as glutamate responses, synaptic density, and neural net integration. We provide here a detailed protocol for performing these cultures and suggest that this method may be highly effective for any neuronal culture that already performs in B27/Neurobasal media.

## Materials and Methods

(Important technique tips are provided in brackets).

### Substrate Preparation

**Figure 1:**
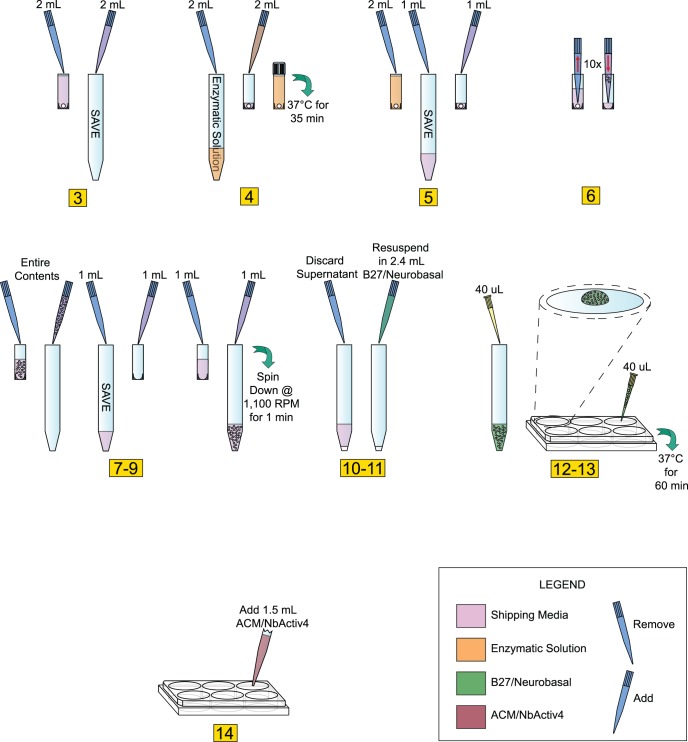
Protocol to Prepare Hippocampal Neuronal Cultures. Diagrammatic representation of the protocol used for preparing 120 DIV E18 hippocampal cultures (see Methods section for details in performing the pictured protocol).

1. On the day of plating, prepare 25 mm coverslips by removing them from 70% ethanol storage solution and propping them up at an angle in each well of a 6-well culture plate to allow drying. *[No more than 5 plates (30 coverslips) should be dried simultaneously for 15–25 minutes in culture hood to avoid over-drying.]*
2. Once dry, shake slips down flat into their respective wells and coat with 1 mL 0.1% poly-D-lysine, taking care to form a liquid meniscus on each slip. Carefully transfer coverslips into incubator, taking care to preserve meniscus. Incubate for 1 hr at 37°C. *[Keeping poly-D-lysine meniscus on top of coverslip is important; this serves to avoid poly-D-lysine coating under coverslip surface that may lead to problematic flotation of coverslip.]*
3. After incubation, remove poly-D-lysine and rinse each coverslip three times with 2 mL sterile deionized water. Take care to ensure coverslips do not completely dry at any point during the rinse. After the third and final rinse, leave coverslips in 2 mL sterile deionized water for at least 1 hr. Remove water just before plating, again, make sure to avoid over-drying. *[This critical step requires attention. Take care to aspirate off all sterile water remaining from the final rinse, but also use caution as not to over-dry the coverslips. Ultimately, the coverslip must be mostly dry as to allow for the meniscus formation during plating (* *Fig. 1*, *Step 12), whereas over-drying can result in the neurons peeling off the glass coverslips days to weeks after plating.]*

### Preparation of Isolated Neurons

(Numbers in Fig. 1 correspond to numbers below).

1. Store tissue at 4°C until ready to use. If dissecting your own cultures, upon isolation of the tissue, store in an appropriate storage media.
2. When ready to plate, make 2 mL of enzymatic solution without B27. In our case, we used Hibernate E-Ca, containing 4 mg (2 mg/mL) of papain. If making your own solution, use a commercially available papain dissociation kit. Make sure to sterile filter solution with 0.2 micron filter after adding papain if source of enzyme is not sterile.
3. Remove the storage media from the dissected tissue and transfer into sterile 15 mL screw-cap tube; be careful not to disturb or remove tissue from original tube. Save the storage media, do not discard.
4. Add 2 mL of media made in Step 2 to tissue (in our case, Hibernate E-Ca containing 2 mg/mL of papain). Incubate for 35 min at 37°C. *[Be sure to add Hibernate E-Ca containing papain slowly as to avoid disturbing tissue.]*
5. Remove enzymatic solution from tissue, again, take care not to disturb or remove tissue. Add back 1 mL of storage media saved in 15 mL tube.
6. Using a 1 mL pipettor with a sterile *plastic* pipette tip (tissue can adhere to glass pipettes), aspirate the tissue with the medium into the pipette and immediately dispense contents back into same container. Take care not to create bubbles. *[This is another critical step that requires attention. Take care to make sure pipette tip remains in a stable position (as shown in* *Fig. 1*, *Step 6). Maintain slow, steady speed when both drawing in and re-dispensing media containing tissue.]*
7. Repeat this trituration step 10–12 times or until most all the tissue is dissociated and the cells are dispersed. *[Under close examination cell dispersion is highly visible. Stop pipetting immediately upon cell dispersion.]*
8. Slowly transfer contents of the tissue tube into a new sterile 15 mL screw-cap tube.
9. Use the remainder of storage medium saved in Step 3 and rinse the interior of the tissue tube before adding it to the sterile 15 mL screw cap tube containing dispersed cells from step 7. *[This step helps ensure minimal wastage, as any remaining cells should be saved with this extra rinse.]*
10. Spin dispersed cells at 1,100 rpm (200Xg) for 1 min.
11. Discard the supernatant while being careful not to remove any of the cells from cell pellet.
12. Flick tube a few times to loosen the cell pellet. Re-suspend pellet in 2.4 mL of pre-warmed B27/Neurobasal/0.5 mM glutamine medium. Re-suspend by gently pipetting up and down. For E18 Hippocampus, medium includes 25 *µ*M glutamate.
13. Plate cells within a meniscus (approx. 10 mm diameter) at a minimum of 40 *µ*L per 25 mm coverslip. Take care not to disturb meniscus. *[Periodically pipette up and down throughout plating process (no more than once every plate per 6 coverslips) to help maintain equal cell density. Again, plating with meniscus formation is critical.]*
14. Incubate plated cells at 37°C with 5% CO_2_ and/or 9% or 20% oxygen for 1 hr.
15. Add 1.5 mL per well of pre-warmed 1∶1 ACM/NbActiv4. *[Slow and steady media addition rate and proper pipette position are necessary for successful plating density consistency. Position pipette tip at 45° angle along middle of 6-well interior sidewalls, dispense 1.5 mL as slowly and steadily as possible (see Steps 1–14).]*
16. Incubate cells at 37°C with 5% CO_2_ and/or 9% or 20% oxygen.
17. Add Cytosine β-D-arabinofuranoside (Ara-C) to a final concentration of 5 *µ*M, 5–6 days after plating to curb glial proliferation. *[Remove 1/3 of media from each well and replace with equal volume containing final concentration of Ara-C]*
18. After 4 days or longer, neurons are well differentiated. If further culture is desired, change 1/3 of medium with fresh, pre-warmed 1∶1 ACM/NbActiv4 every 7–8 days.

### Materials Needed

- Poly-D-lysine (Sigma P6407) for substrate
- Papain (Sigma P4762) for enzymatic dissociation
- Sterile *plastic* pipette tips
- Sterile centrifuge tubes
- Centrifuge to operate at 200×g
- Water bath at 37°C
- General cell culture supplies
  - ○ 6-well culture plates
  - ○ 25 mm round coverslip glasses, #1 thickness (Warner Instruments cat. no. CS-25R)
- Additional media
  - ○ Hibernate E-Ca (BrainBits SKU: HE)
  - ○ Neurobasal Medium (cat. no. 21103-049)
  - ○ B-27 supplement (Invitrogen cat. no. 17504-044)
  - ○ Glutamine/GlutaMAX (Invitrogen cat. no. 35050-061)
  - ○ ACM (ScienCell cat. no. 1811)
  - ○ NbActiv4 (BrainBits product code: NbActiv4 500)
  - ○ Cytosine β-D-arabinofuranoside (Sigma C1768)

### Antibodies and Reagents

Fura-2, AM, Alexa Fluor 488 goat anti-rabbit IgG, Alexa Fluor 647 donkey anti-goat IgG, Alexa Fluor 546 goat anti-mouse IgG, Alexa Fluor 647 goat anti-chicken IgG, and ProLong Gold antifade reagent were from Molecular Probes. Chicken IgY MAP-2 antibody was from Neuromics. Rabbit polyclonal anti-GFAP and mouse monoclonal anti-β-catenin antibodies were from Abcam, Inc. Rabbit polyclonal Synapsin antibody was from Millipore. Mouse S100B and rabbit βIII-Tubulin antibodies were generous gifts from Daniel Daugherty of the Winben Deng Lab (University of California, Davis, School of Medicine). Rabbit polyclonal IP_3_R antibody was a generous gift from Dr. Darren Boehning (University of Texas Medical Branch). Wnt3a and Wnt5a ligands were obtained from R&D systems; L-glutamine was from Cellgro; goat serum was from Gibco; L-glutamic acid, bovine serum albumin, and saponin were from Sigma; NaCl was from VWR; KCl was from EMD Chemicals; MgCl_2_θ·6H_2_O, glucose, and HEPES were from J.T. Baker; paraformaldehyde was from Mallinckrodt Chemicals. Lipofectamine 2000 and Opti-MEM were from Life Technologies. Papain Dissociation System was from Worthington Biochemical Corporation.

### Intracellular Calcium Measurements

The methodology for culture and cytosolic Ca^2+^ measurements within rat E18 hippocampal neurons using fura-2 were as described previously [11]–[13]. Briefly, cells were grown on glass coverslips as described above and fura-2-loaded by incubation with 2 *µ*M fura-2, AM in HEPES-buffered saline solution with CaCl_2_ and L-glutamine (107 mM NaCl, 7.24 mM KCl, 1.18 mM MgCl_2_·6H_2_O, 2 mM CaCl_2_, 11.5 mM glucose, 20 mM HEPES, 2 mM L-glutamine, pH 7.2, 0.1% bovine serum albumin) for 30 min at 37°C. After de-esterification in fresh loading medium for 30 min at room temperature, coverslips were inserted into a Dvorak-Stotler Controlled-Environment Culture Chamber (DSC200, Nicholson Precision Instrument, Gaithersburg, MD). Groups of 40–100 individual fura-2-loaded cells were viewed through a Nikon Eclipse TS100 microscope and Plan Fluor 20X/0.50 objective. Fluorescence emission at 510 nm was monitored at room temperature using an Intracellular Imaging Inc. InCyt Standard IM Fluorescence Imaging System with excitation at 340 and 380 nm. Emission ratios were converted to [Ca^2+^ nM] using a standard curve generated using Ca^2+^ standards. Resting Ca^2+^ levels were approximately 200–250 nM; maximal activation by glutamate resulted in up to 2400 nM Ca^2+^.

### Immunocytochemistry

Rat E18 hippocampal neurons were grown on coverslips for 15–120 days, washed three times in 1X PBS for 15 min, fixed with 3% paraformaldehyde in 2X PBS for 30 min, and washed an additional three times in 1X PBS for 15 min. Cells were permeabilized in saponin solution with goat serum (2X PBS, 1% bovine serum albumin, 1% goat serum, and 0.2% w/v saponin) for 1 hr at room temperature. Coverslips were subsequently inverted on 250 *µ*L of primary antibody in saponin solution (MAP-2 antibody 1∶7,500, GFAP antibody 1∶10,000, Synapsin antibody 1∶10,000, S100B antibody 1∶10,000, βIII-Tubulin antibody 1∶300, IP_3_R antibody 1∶1,000 and β-catenin antibody 1∶1,000) in a wet chamber overnight at 4°C. Coverslips were washed three times in 2X PBS for 15 min and inverted on 250 *µ*L of secondary antibody in saponin solution (Alexa Fluor 488 goat anti-rabbit 1∶1,000, Alexa Fluor 647 donkey anti-goat 1∶1,000, Alexa Fluor 546 goat anti-mouse 1∶1,000, and Alexa Fluor 647 goat anti-chicken 1∶1,000) for 1 hr at room temperature. Finally, coverslips were washed three times in 2X PBS for 15 min and mounted onto slides with ProLong Gold antifade reagent. Fluorescence signals were detected with a Zeiss LSM 510 confocal laser scanning microscope and Zeiss LSM 4.3 software, using Zeiss Plan-APOCHROMAT 10X/0.45, Zeiss Plan-NEOFLUAR 40X/1.30 Oil, and Zeiss Plan-APOCHROMAT 63X/1.40 Oil objectives.

### Bright Field Microscopy

Bright field images were taken using a Zeiss Observer.Z1 microscope with an EC PlanNeo 10X/0.30 Ph1 objective, 2.5X optovar, and Photometrics QuantEM:512SC camera.

### Transfection

Transfection was performed as previously described [14]. The following briefly describes the process as performed 4 days after cell seeding. 8 *µ*L of Lipofectamine 2000 were diluted in 100 *µ*L Opti-MEM and briefly vortexed while 1.5 *µ*L YFP vector was combined with 100 *µ*L Opti-MEM and briefly vortexed. After each tube was left to incubate at room temperature for 5 minutes, they were mixed, vortexed, and incubated for an additional 10 minutes. The 1.5 mL (per well) of ACM/NbActiv4 was removed and replaced with 800 *µ*L of Opti-MEM, in which the previously incubated Lipofectamine/DNA complex was added. The removed media was saved, and mixed with fresh ACM/NbActiv4 at a 1∶1 ratio. After 4–6 hours, the transfection media was replaced with the 1.5 mL of the previously prepared ACM/NbActiv4. Images were obtained using a Nikon Eclipse TS100 microscope and Plan Fluor 10X/0.25 Ph1 ADL objective. Fluorescence emission at 510 nm was observed at room temperature using an Intracellular Imaging Inc. InCyt Standard IM Fluorescence Imaging System with excitation at 485 nm.

### Animals

P0 California mouse (*Peromyscus californicus*) neonates (n = 2) were euthanized by rapid decapitation. Brains were rapidly removed and dissected to remove hippocampus and cortex. Tissue was homogenized using a papain dissociation kit (Worthington) according to the manufacturer’s instructions. Tissue was kept on papain at 37°C for 1.5 hr. Following dissociation, brain cell cultures were maintained as described above. Animals were treated according to procedures approved by the UC Davis Institutional Animal Care and Use Committee and which adhered to the National Institutes of Health Guide for the Care and Use of Laboratory Animals.

## Results and Discussion

### Substrate and Media

During the optimization of this protocol, several steps were identified as critical for achieving our desired results, including the use of ACM/NbActiv4 media, German glass coverslips as a substrate, and Ara-C for properly curbing glial proliferation. After assessing many different combinations and various ratios of media types (N2 media, Neurobasal media, ACM, NbActiv4, etc.), we determined that a 1∶1 ratio of ACM and NbActiv4 was most conducive to neuronal maturation, differentiation, and longevity. In accordance with the Banker method, we performed multiple trials comparing coverslips made from different glass sources and determined empirically that coverslips manufactured from German borosilicate glass had a significantly higher rate of neuronal attachment and survivability versus coverslips obtained from domestic sources. Lastly, we determined that the addition of 5 µM Ara-C after 5–6 days of culturing was a suitable time frame for optimal retardation of glial proliferation; however, depending on the application, this time could be extended or reduced.

### Maturation and Differentiation

**Figure 2:**
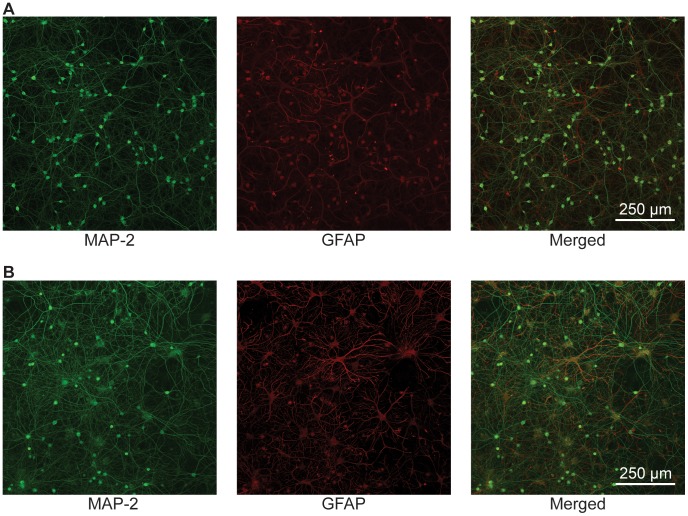
10X Confocal Images Comparing 15 and 120 DIV Hippocampal Cultures. A series of confocal laser scanning microscope images of 15 DIV (A) and 120 DIV (B) hippocampal neuronal cultures. Cultures were immunostained for MAP-2 (green) and GFAP (red). Immunofluorescence detection was performed via confocal microscope using a 10X objective. The 120 DIV cultures displayed an increase in field diameter, dendritic arborization, and an overall denser neural network compared to the 15 DIV cultures. Although glial processes increased with age, glial population was maintained between 10 and 20% of the entire co-culture. Unlike previous attempts at long-term cultures, the persistence of MAP-2 staining and absence of varicosities illustrates limited degradation of dendrites, even after 120 DIV.

Figure 2 depicts representative laser scanning confocal images of E18 hippocampal cultures grown for 30 (A) and 120 (B) days *in vitro* (DIV) using the method described above. The 120 DIV image depicts the longest culture maintained in the literature to date by more than 60 days. Under these conditions, we are able to maintain a stable glia to neuron ratio (10–20% glia) over the course of our cultures. Thus, as consistent with previously described long-term hippocampal cultures, we observe increased arborization, but not proliferation, of both glia and pyramidal cells when compared to younger cultures [6]. However, unlike earlier described long-term hippocampal cultures we do not observe the same decrease in MAP-2 expression, which has been previously reported [6]. This is likely due to the improved health of these cultures.

### Synaptic Density

**Figure 3:**
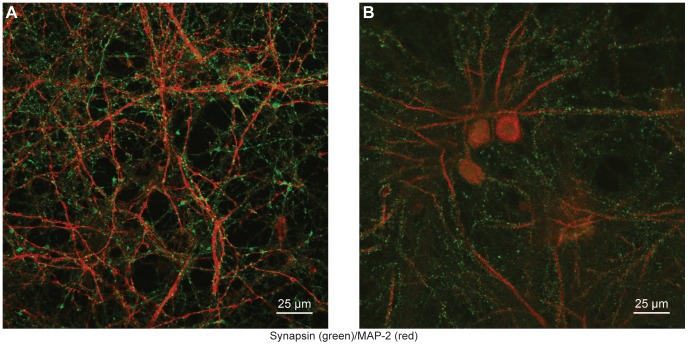
Synaptic Density in 15 and 120 DIV Hippocampal Cultures. A confocal laser scanning microscope image of 15 (A) and 120 DIV (B) E18 hippocampal cultures at 40X magnification. Cultures were immunostained for MAP-2 (red) and Synapsin (green). In comparison to 15 DIV, the 120 DIV cultures display a higher density of synaptic puncta. While overall synapse count is greater in the 15 DIV image (2,201 versus 1,898) there is a corresponding increase in total neurite area compared to the 120 DIV image. Thus, when synapse count relative to total neurite area is normalized the synapse to neurite ratio remains greater for the 120 DIV cultures (0.424 versus 0.364), demonstrating a higher synaptic density.

Figure 3 demonstrates the synaptic density achieved with these culturing techniques. Quantification of these cultures reveals an increase in total synapses per unit length of dendritic processes in older cultures when comparing 15 and 120 DIV samples. On average 15 DIV cultures maintained 1.00 synapses per micrometer while 120 DIV cultures averaged 1.29 synapses per micrometer (with *P*<0.05). This is in strong correlation to the 1.10 total synapses per micrometer reported in previous studies of 14 DIV cultures [15]. However, unlike findings from Boyer et al. (1998) where total synapse numbers decrease after 14 DIV, our cultures show an increase in synaptic puncta per unit length of dendrite over time. Furthermore, our 120 DIV results are closer in proximity to estimates of 1.7 spine synapses per micrometer in the adult rat hippocampus [16]. Additionally, we used ImageJ software (NIH) to quantify relative synapse to dendrite area and found that 120 DIV cultures showed a 16.5% increase over 15 DIV cultures (0.424 versus 0.364 respectively).

### Tripartite Synapse

Previous studies underline the importance of the contribution of glia in the development and function of synapses [9], [17]–[21]. It has been shown that individual astrocytes can contact up to 140,000 synapses [22]. In addition to diffusible secreted factors, direct astrocytic contacts have proven do induce large-scale maturation and a subsequent up-regulation of synapse formation in neurons [23]. Indeed, astrocytic membrane-bound ligands such as ephrin-A3 regulate spine morphology in pyramidal cells of the hippocampus, and as a whole, may contribute to initial synapse formation [24]. Additionally, successful labeling has revealed intricate interactions between astrocytes and dendritic arbors of CA1 neurons [25]. These interactions have been directly associated with synaptic plasticity, where astrocytes release glutamate and play a large role in regulating synaptic transmission [26]–[29].

**Figure 4:**
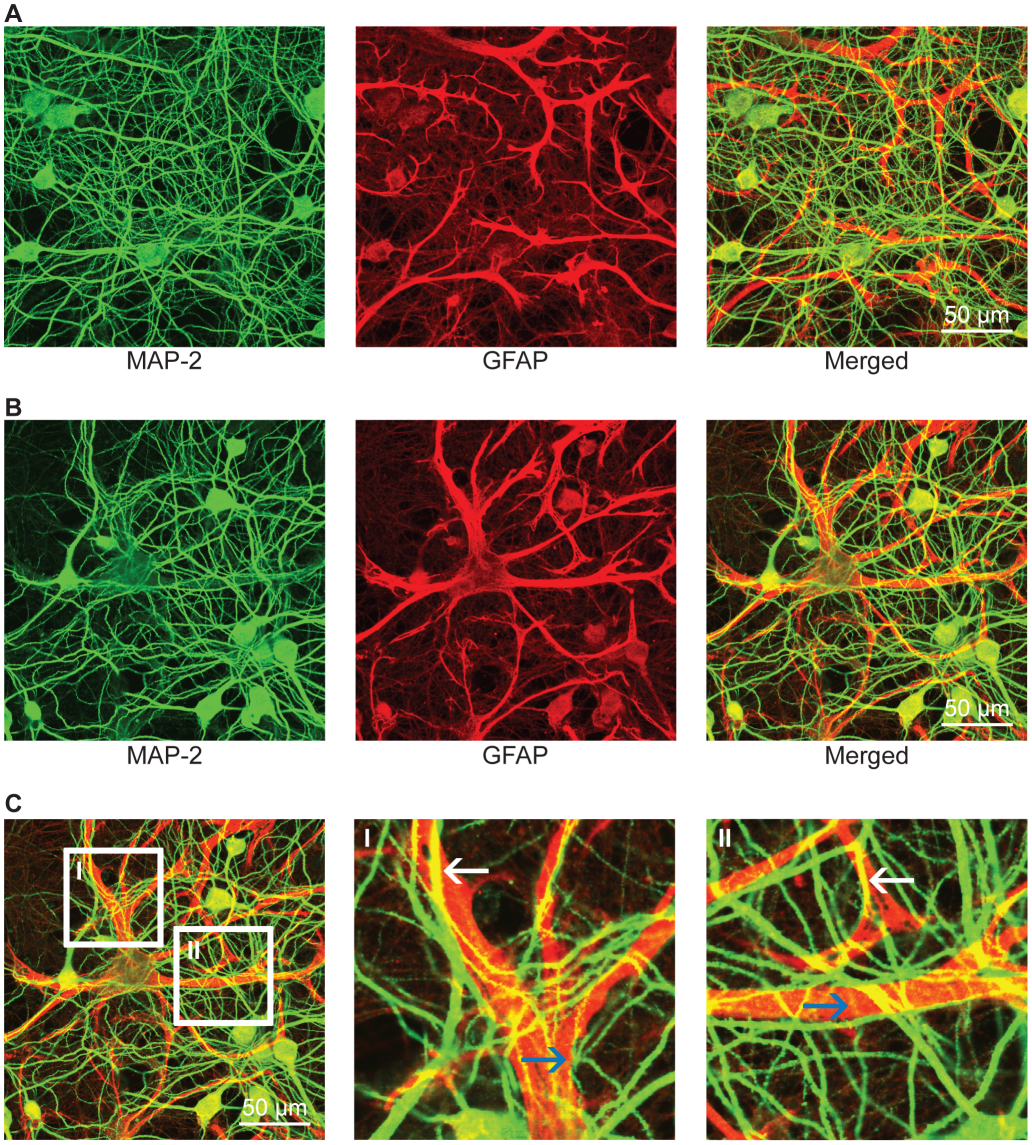
40X Confocal Images of 30 DIV Hippocampal Cultures. Immunofluorescence detection of MAP-2 (green) and GFAP (red) in 30 DIV (A–C) cultured E18 hippocampal cells using a 40X objective. These images (A and B) clearly depict the intimate physical contact between glia processes and dendritic arbors. Under closer examination (CI and CII), it is clear that the dendrites have grown both bellow (blue arrows) and above (white arrows) glial processes, forming a highly interconnected three-dimensional network by 30 DIV.

**Figure 5:**
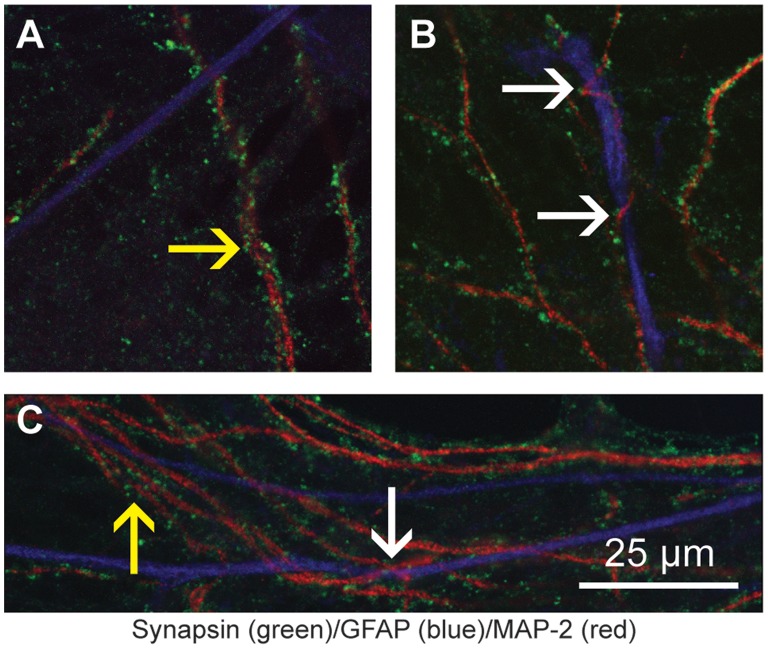
63X Confocal Images of Tripartite Synapses After 120 DIV. A confocal laser scanning microscope image of 120 DIV E18 hippocampal cultures at 63X magnification. Cultures were immunostained for MAP-2 (red), Synapsin (green), and S100B (blue). Yellow arrows (A and C) indicate synapse-rich dendrites. White arrows (B–C) indicate where dendritic shafts entwine around glial processes.

For reasons mentioned above, it is important to preserve intimate contacts between astrocytes and neurons during culturing. Figure 4 illustrates a multi-layered culture with neuronal/glial contacts resembling those observed in tissue slices. This figure depicts the direct interaction between pyramidal dendrites stained with anti-MAP-2 (green), and glial processes stained with anti-GFAP (red), consistent with observations reported by Nishida et al. (2007) [24]. Careful examination reveals intricate associations where dendrites appear to entwine glial processes (see Fig. 4 and Fig. 5). In addition, Figure 5 depicts glial projections (blue), as stained by S100B, closely associated with synapse-rich dendrites (red) as stained by MAP-2. This crucial interaction cannot be achieved using the conventional Banker method due to the physical separation of neuronal and glial cultures.

### Intracellular Calcium Measurements

**Figure 6:**
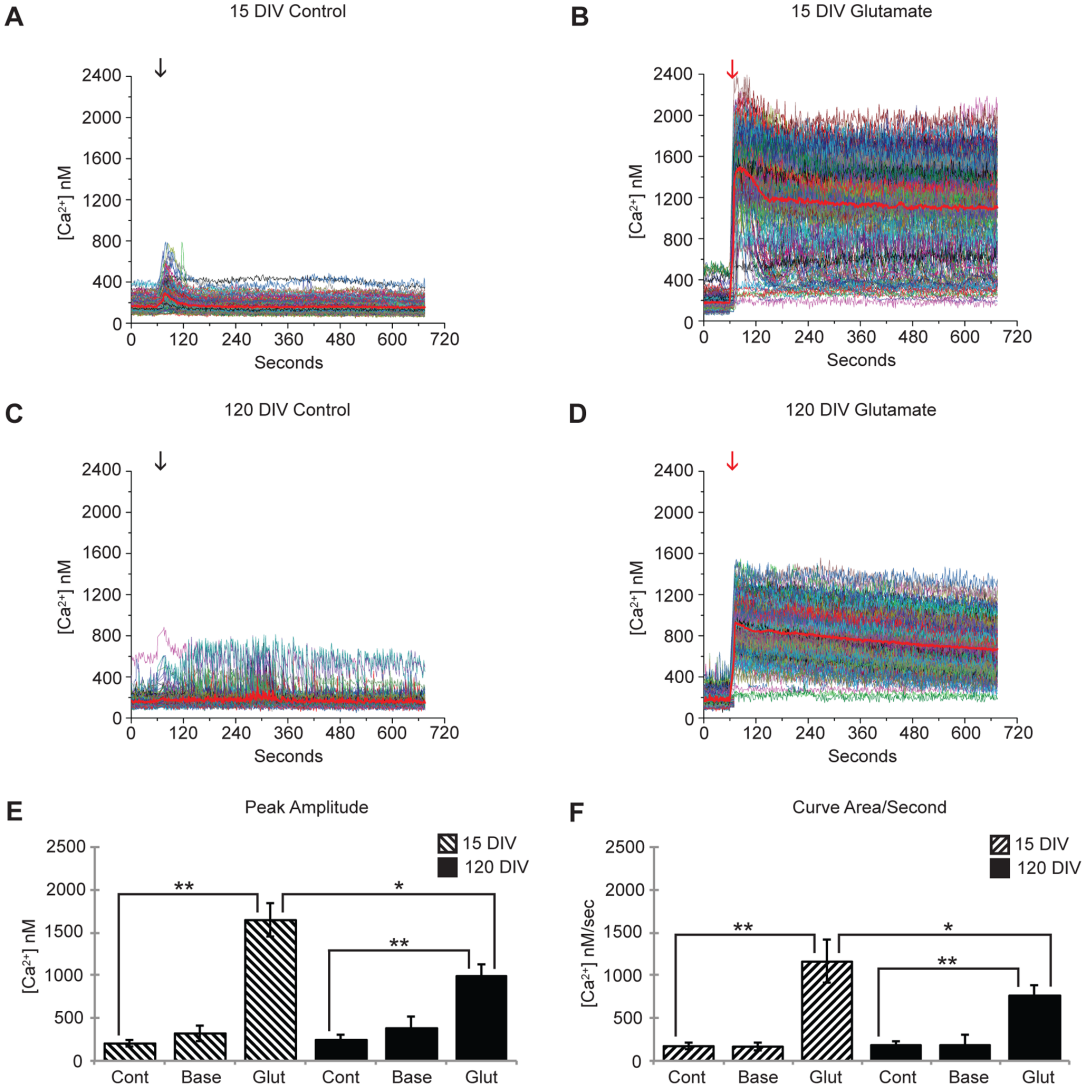
Intracellular Measurements During Glutamate-Induced Calcium Flux. (A,B) 15 DIV and (C,D)120 DIV calcium imaging experiments. Baseline measurements were taken during the first 60 seconds in HEPES-buffered saline solution with 2 mM CaCl_2_ and 2 mM L-glutamine, followed by a solution swap of identical buffer [black arrows] (A,C) for controls, or HEPES-buffered saline solution with 2 mM CaCl_2_, 2 mM L-glutamine, and 1 *µ*M glutamate [red arrows] (B,D). (E) Peak amplitude and (F) curve area per second values are means ± SE collected from 10 different experiments each comprising of approximately 100 cells. * and ** indicate significant differences between mean responses with *P*<0.05 and *P*<0.01, respectively, using 2-tailed paired student *t*-test. Notice glutamate response for 120 DIV cultures is significantly lower for the 15 DIV cultures, while baseline calcium levels are not significantly different from calcium control methods.

To ensure that these cultures were comprised of viable neurons, we performed calcium imaging experiments and observed a glutamate-induced increase in intracellular calcium concentration over nearly all of the sampled cells (Fig. 6). Consistent with previous results comparing age-related calcium changes in rat hippocampal brain slices, the addition of 1 *µ*M glutamate elicited a significantly larger calcium flux in 15 DIV cultures compared to those of 120 DIV [30]. These results further elucidate the degree in which these long-term cultures appear to mirror neuronal organoid activity.

**Figure 7:**
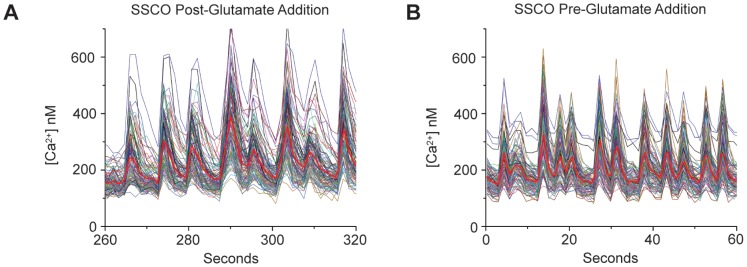
Spontaneous, Synchronous Calcium Oscillations (SSCOs). Intracellular calcium measurement of oscillatory calcium transients during glutamate treatment (A) and baseline Ca^+^ recording (B). Solid red line indicates the average [Ca^2+^ nM]_i_. Both graphs represent a population of 99 individual pyramidal cells and demonstrate highly coordinated activity between cells within the field.

These long-term *in vivo* cultures also form complete large-scale neuronal circuits that produce spontaneous, synchronous calcium oscillations (SSCOs) that can persist throughout the imaging field during both baseline and excitatory phases of intracellular calcium measurements (Fig. 7). It has been previously demonstrated that SSCOs are dependent on the establishment of functional synaptic contacts, and that in all cases, an extensive network of synaptic contacts were required for this oscillatory phenomenon to occur. Moreover, it has recently been shown that glial populations present in hippocampal cultures influence the total level of synchronization, and so, again, this indicates a specific interdependence between the glial and neuronal networks [31]. These intracellular calcium oscillations represent an example of typical physiological interactions in neuronal circuits, and are the result of intense synaptic activity [32]. SSCOs observed in these experiments represent yet another example of the viability and physiologically accurate nature of these long-term cultures.

### Growth and Development

**Figure 8:**
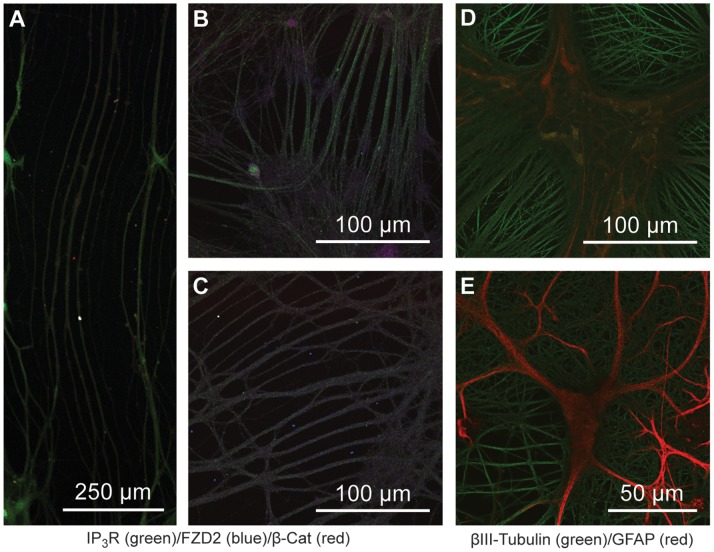
Confocal Images Demonstrating Organized Growth Patterns. A series of confocal laser scanning microscope images of E18 hippocampal cultures; (A) 67 DIV at 10X magnification, (B–C) 80 DIV at 40X magnification, (D) 120 DIV at 40X magnification, and (E) 40 DIV at 63X magnification. Cultures imaged in (A–C) were fixed and immunostained for IP_3_R (green) and β-catenin (red) while (D–E) were fixed and immunostained for βIII-Tubulin (green) and GFAP (red). The 67 DIV cultures displayed very long parallel neurite formations (millimeters in length) not seen in younger cultures (A). These formations continue to expand and can form more robust neurites as can be seen at 40x magnification (B–C). When looking at neuron specific staining (β III-Tubulin), one can find many interesting patterns of neurite formation including parallel growth (D) or grid-like patterns (E).

Figure 8 is a representative confocal image of older cultures imaged using confocal microscopy at various magnifications (10–63X). These cultures have a propensity to grow neurites that are reminiscent of parallel fibers found in the brain (Fig. 8A). We have only observed this particular type of growth to occur after 30 days in culture, and more regularly after at least 50 days in culture. In magnified views (Fig. 8B–C), we observe a high degree of order and remarkable growth patterns. These elaborate patterns and long stretches of parallel growth suggest some form of communication throughout neurite development. This idea is strengthened by examining neuron-specific immunostaining (βIII-Tubulin), where neurites appear to form complex matrix patterns (Fig. 8E). It is also worth noting that the neurite network continues to increase in overall density and field depth, as can be seen by comparing 40 DIV (Fig. 8E) to 120 DIV (Fig.8D), where the neurites have developed into an additional network layer above glial processes. As a result, we found that as cultures age, the neurite field continues to increase in overall diameter and depth.

**Figure 9:**
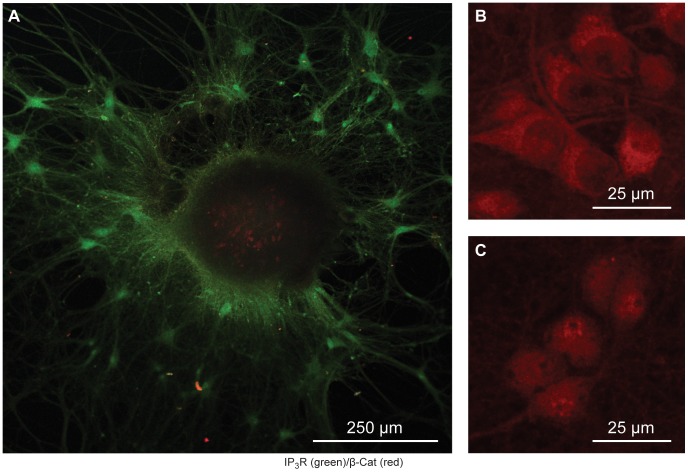
Hippocampal Neurosphere. Confocal laser scanning microscope images captured from (A) 67 DIV E18 hippocampal culture at 10X magnification and (B–C) close-up view of 14 DIV E18 hippocampal culture originally at 40X magnification. (A) Culture was immunostained for IP_3_R (green) and β-catenin (red). Differential staining patterns suggest multiple cell types within the neurosphere. Note the localization of β-catenin within the center of the cellular mass. As a positive control for our β-catenin staining, 14 DIV cultures were treated with Wnt5 (B) and Wnt3 (C) prior to fixing and staining to re-localize β-catenin either in the cytoplasm (Wnt5) or in the nucleus (Wnt3). Notice that the β-catenin (red) is more dominant in the nucleus after treatment with Wnt3 (C) versus Wnt5 (B), demonstrating that the β-catenin staining is highly specific.

**Figure 10:**
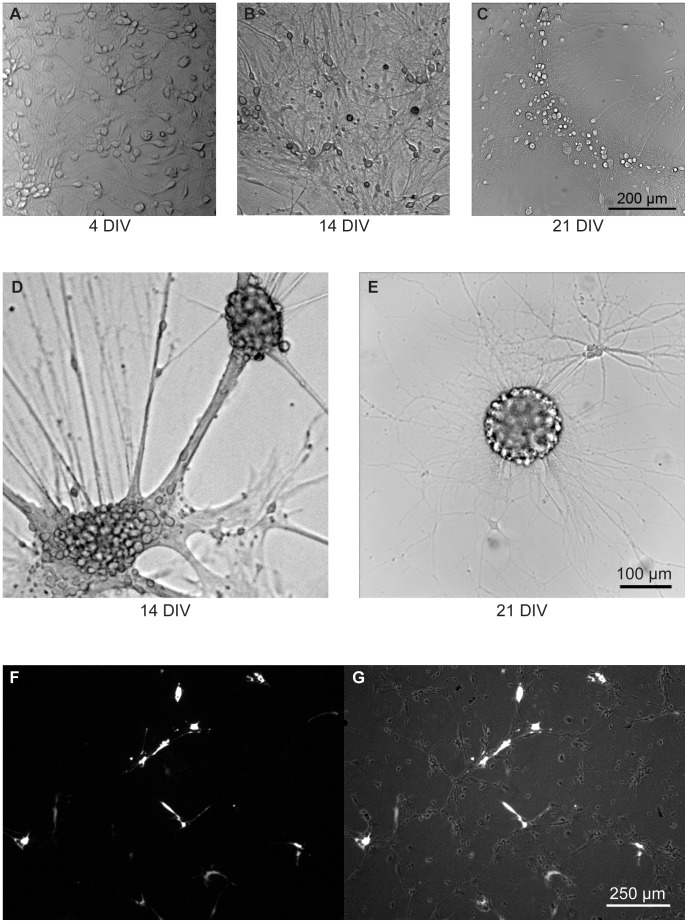
P0 California mouse Bright Field Images. A series of bright field images taken from P0 California mouse hippocampus (A–E) and cortical (F–G) regions. Hippocampal images were taken at 4 (A), 14 (B), and 21 (C) DIV. Notice the spatial rearrangement progression from day 4 to day 21. Neurospheres were detected after day 7, and are shown at 14 days (D) and 21 days (E). Cortical neurons shown at 9 DIV (F–G) were transfected with YFP at 10–15% efficiency.

These observations suggest that these cultures are significantly different in their cellular functions at these disparate time points, which is consistent with the calcium imaging results mentioned above. Further studies monitoring SSCOs along with different levels and types of neurite development are needed to characterize these associations. In addition, future studies are needed to determine what types of co-cultures and/or organoid precursors can be created using this method. Of note, we have observed a propensity of these cultures to produce what appears to be neurospheres with a high degree of proliferation (Fig. 9,10D–E). Indeed, the neurosphere-like structures we observed from both the rat E18 hippocampal neurons (Fig. 9) and the P0 neonatal California mice hippocampal neurons (Fig. 10D–E) appear very similar to those reported in the literature [33]. Further, they accord with sizes reported and levels of protein expression. As shown in Fig. 9A, the center of the neurosphere stains positive for β-catenin (red), while the migrating cells appear to express much lower levels. This is consistent with previous studies demonstrating the requirement of β-catenin expression for obtaining developing and adherent neurospheres [34], [35]. As a positive control for β-catenin staining, we stimulated our neurons with Wnt ligands, which promote β-catenin re-localization to the cytosol (Wnt5, Fig. 9B), or the nucleus (Wnt3, Fig. 9C).

### Other Types of Neuronal Cultures

Given these results, we assessed whether our protocol could be used successfully in multiple neuronal types from a different, but related laboratory animal model species. The California mouse is an undomesticated species that is more closely related to rats than mice (*Mus musculus).* The lack of domestication makes them useful in studies that examine environmental impacts on physiology and behavior. When we applied our method to both the hippocampus and cortex from P0 neonates of this species we observed that both of these neuronal populations performed well. We observed dramatic expansion and the initiation of migration within the hippocampal neuronal populations over 21 days (Fig. 10. A-C). In addition, the cortical neurons were transfected using a standard Lipofectamine 2000 transfection protocol (see Methods). When cortical neurons are transfected 4 days post-plating we obtain 10–15% transfection efficiency of both glia and neurons (Fig. 10 F-G) as observed at day 9. The efficacy of our cell culture protocol in both a domesticated species like the rat and undomesticated California mouse suggests that the method could apply to broad areas of research that utilize specialized animal models.

## Conclusion

Given the above results, as a whole, we suggest that this method provides an efficient, cost-effective, and scalable method for producing both short- and long-term hippocampal cultures that have significant advantages over current techniques, in particular, as regards their developmental progression. Furthermore, this method can be performed in almost any laboratory due to the availability of neuronal dissections from industrial sources. While the limits to which these cultures can replicate *in vivo* physiological responses has yet to be determined, the data presented in this study demonstrates that many of the conventional measurements taken from brain slice studies are closely recapitulated by our cultures. Ultimately, this culturing method results in a physiologically relevant model system that can be cultured for significantly longer periods of time than other long-term hippocampal studies that we are aware of [6], [7]. In short, our cultures represent an *ex vivo* model system that more accurately portrays an *in vivo* hippocampal tissue with respect to glial populations while the primary neurons remain in direct contact with each other, thereby forming physiologically relevant synapses. In closing, we suggest that exploration of this method may allow for the production of true organoid neuronal cultures. Moreover, as hippocampal cultures already serve as an ongoing model for Alzheimer’s disease and in other neurodegeneration studies [6], [36], these cultures may provide distinct advantages when testing the effects of long-term genetic modification and/or long-term pharmacological tests. Our future research will focus on (i) exploring the time limits of growing these neurons and cell types, (ii) the physiological processes that can be measured in these cultures, and (iii) understanding how closely the development of these cultures mirrors *in vivo* development.
